# Unicentric epithelioid hemangioendothelioma of the calcaneus: a case report and review of literature

**DOI:** 10.1186/s13569-018-0092-z

**Published:** 2018-04-06

**Authors:** Mark C. Plumby, Patrick Bacaj, Brock A. Lindsey

**Affiliations:** 1 Department of Orthopaedics, West Virginia University, PO Box 9196, Morgantown, WV 26506-9196 USA; 2 Department of Pathology, West Virginia University, PO Box 9203, Morgantown, WV 26506-9203 USA

**Keywords:** Hemangioendothelioma, Epithelioid, Angiosarcoma, Vascular tumor

## Abstract

**Background:**

This review of the literature combined with a clinical case will allow the illustration of *a favorable outcome* of this variable *low grade* malignancy, display a role for limb salvage surgery with intralesional treatment, and offer a clinical example of epithelioid hemangioendothelioma, a rare malignancy.

**Case presentation:**

The case report presents a case of solitary epithelioid hemangioendothelioma (EHE) of the calcaneus in a 60-year-old male. Primary vascular tumors of the bone are rare; however, EHE is one of the most common primary malignant vascular tumors to occur in bone. A review of the literature found few cases that involved the calcaneus; those cases found that involved the calcaneus were either part of a multifocal or metastatic disease process. Our case *presents a 45*-*month clinical follow*-*up* of solitary EHE in the calcaneus treated with surgical excision by curettage and cementing.

**Conclusion:**

This case has clinical follow-up greater than 2 years post-operatively and could be a guide for treatment of a rare disorder with a substantial paucity of literature.

## Background

Most lytic lesions of bone are a result of metastatic disease; primary bone tumors are far more uncommon. Benign primary bone entities are more common than their malignant counterparts. It was estimated that the age adjusted incidence rate for all bone and joint malignancies is 0.9 per 100,000 persons/year [1]. Epithelioid hemangioendothelioma (EHE) is a vascular tumor that has a propensity to occur in bone. Among vascular tumors, it is considered a *low grade malignancy*, between hemangioma and angiosarcoma [2–6]. The low prevalence for primary malignant bone tumors, and thus vascular tumors of bone, has limited the extent of our knowledge. However, there has been much work over the last few decades to help adequately identify and classify EHE and its family of vascular tumors [6–13].

EHE was given its name by Weiss and Enzinger in 1982 to reflect that it is neither completely benign nor fully malignant [7]. More recently, it has been said that it is the most common malignant vascular tumor of bone, though still relatively rare, accounting for less than 1% of all bone tumors. It has an overall prognosis that favors survival [13]. EHE has been shown to occur slightly more in males [7, 13] and at any age with a peak in the second and third decades of life [4]. The majority of cases have been shown to occur in the lower extremities, primarily the tibia and femur [4, 7, 9, 13]. The most common complaint is local pain at the site of the lesion. Radiographic imaging typically shows a lytic lesion; diagnosis is made with a biopsy and study of the histopathological and immunohistochemical features.

To date, there have been no reports of solitary EHE in the calcaneus. There are a few cases that show the involvement of the calcaneus, all of which occurred in a multifocal or metastatic setting [5, 14–17]. In this case report, we describe a solitary EHE of the calcaneus that did not appear on initial radiographic imaging.

## Case presentation

**Fig. 1:**
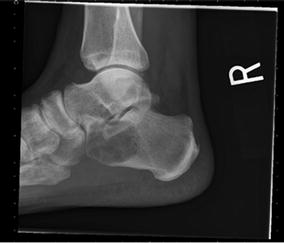
Pre-operative radiograph of large lytic lesion in the calcaneus

**Fig. 2:**
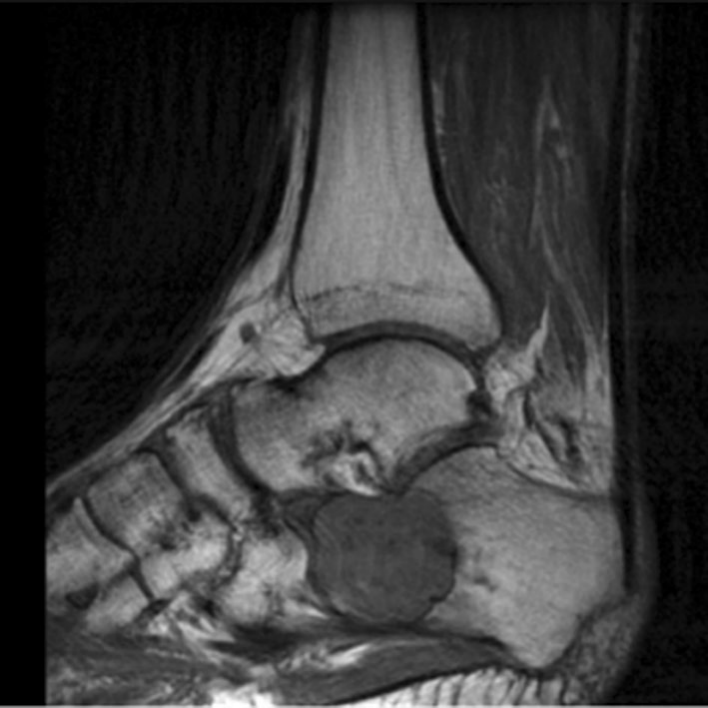
Preoperative sagittal T1 image of the calcaneus showing solid lesion with fairly homogenous signal

**Fig. 3:**
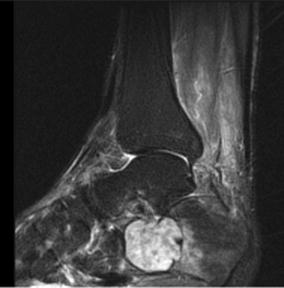
Preoperative sagittal T2 image with some heterogeneity within the lesion surrounding bony edema, sclerotic border around the lesion, but with concern for cortical breakthrough in the subtalar joint

**Fig. 4:**
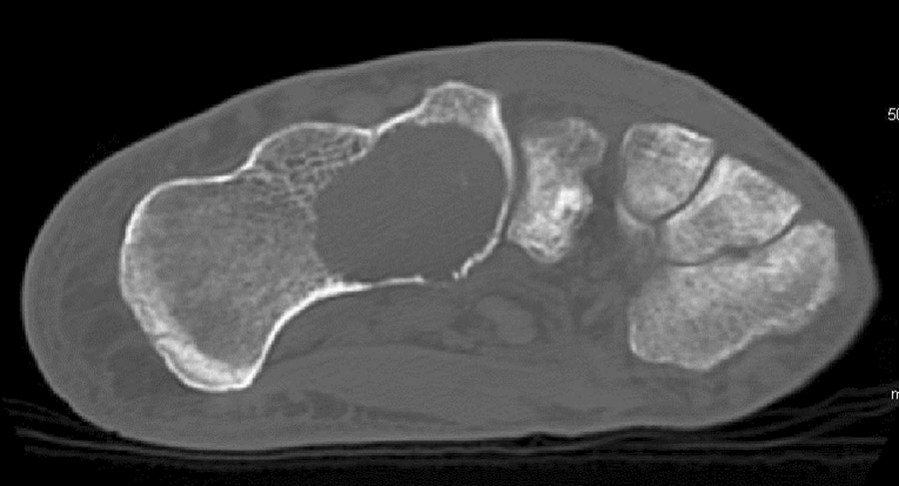
Axial CT scan of lesion preoperatively displaying concern for cortical destruction

**Fig. 5:**
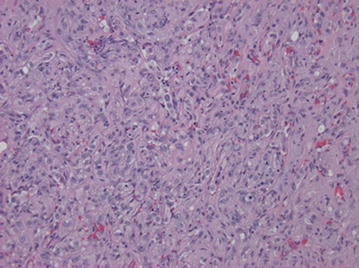
H&E ×200. The tumor consists of large epithelioid and spindle cells arranged in cords, clusters and as single cells within a myxoid to hyalinized stroma

**Fig. 6:**
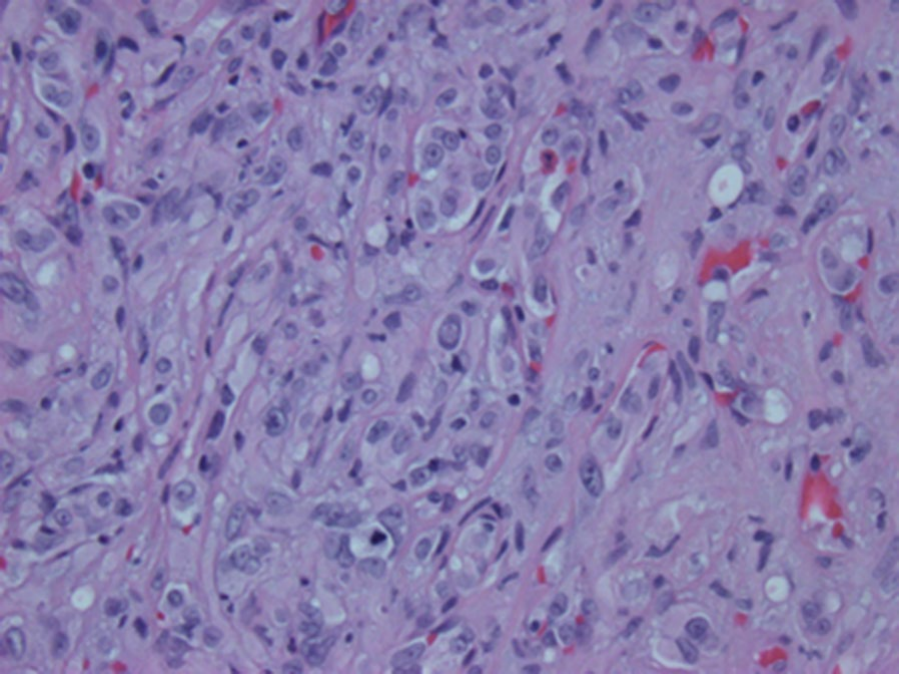
H&E ×400. The tumor cells have moderately abundant eosinophilic cytoplasm with occasional cytoplasmic vacuoles. Nuclei are round with prominent nucleoli

**Fig. 7:**
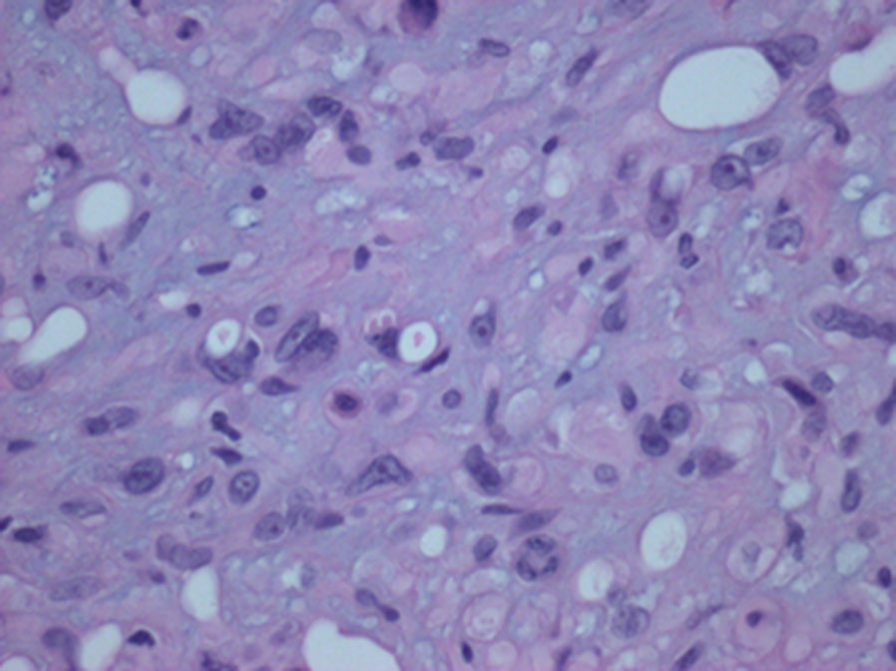
H&E ×600. The vacuoles represent attempts at blood vessel formation and may contain red blood cell fragments

**Fig. 8:**
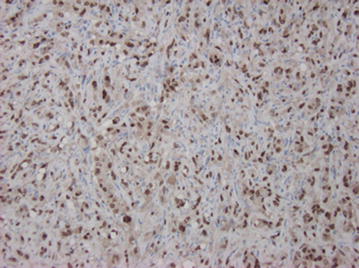
CAMTA1 immunohistochemical stain, ×200. Tumor cell nuclei stain positively with antibody to CAMTA1 protein, a fusion protein unique to this tumor

**Fig. 9:**
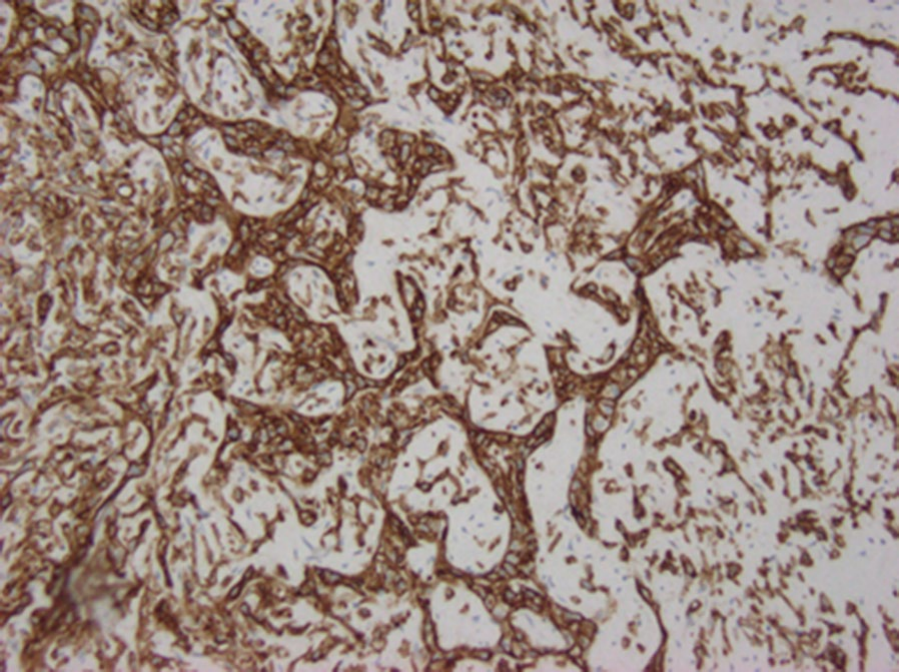
CD34 immunohistochemical stain, ×200. Tumor cells stain positively for endothelial marker CD34. This stain also highlights the anastomosing cord-like architecture of the tumor which recapitulates the formation of primitive blood vessels

A 60-year-old male was evaluated for insidious onset of right foot pain over 4 months, made worse by walking and standing. Radiographs at an outside facility were reportedly normal. Two corticosteroid injections into the sinus tarsus provided no relief. Upon referral new radiographs were obtained (Fig. 1). Magnetic resonance imaging (MRI) showed a complex lesion in the anterior calcaneus with some surrounding edema and potential concern for cortical disruption (Figs. 2, 3). A computed tomography (CT) guided biopsy of the cyst showed a lytic destructive lesion within the central and anterior process of the calcaneus. There was cortical destruction along multiple sites at the margins of the lesion and slight scattered calcifications (Fig. 4). The pathologic report revealed cords and clusters of epithelioid cells and foci of spindle cells in a myxochondroid matrix (Figs. 5, 6). Some of the epithelioid cells contained vacuoles and rare erythrocyte (Fig. 7). Immunohistochemical stains identified CD31, CD34, *and CAMTA1* (Figs. 8, 9). Mixed cytokeratin stain (MCK) showed very focal and equivocal staining and D2-40 and epithelial membrane antigen (EMA) were negative. There was extensive necrosis and some spindling but no invasion of the surrounding bone. These findings supported a diagnosis of epithelioid hemangioendothelioma.

The patient was referred to our orthopaedic clinic roughly 6 months after initial presentation. The right lower extremity was neurovascularly intact with no swelling, masses, or skin lesions. There was mild tenderness to palpation of the calcaneus and no palpable nodes in the popliteal or inguinal regions. At this time, radiographs showed a significant cystic lesion with cortical breakthrough and no evidence of collapse. Routine bloodwork was normal. Positron emission tomography/computed tomography (PET/CT) ruled out proximal metastasis; however, bilateral hilar and mediastinal adenopathy were reported along with increased signal in the stomach and bilateral parotid glands.

**Fig. 10:**
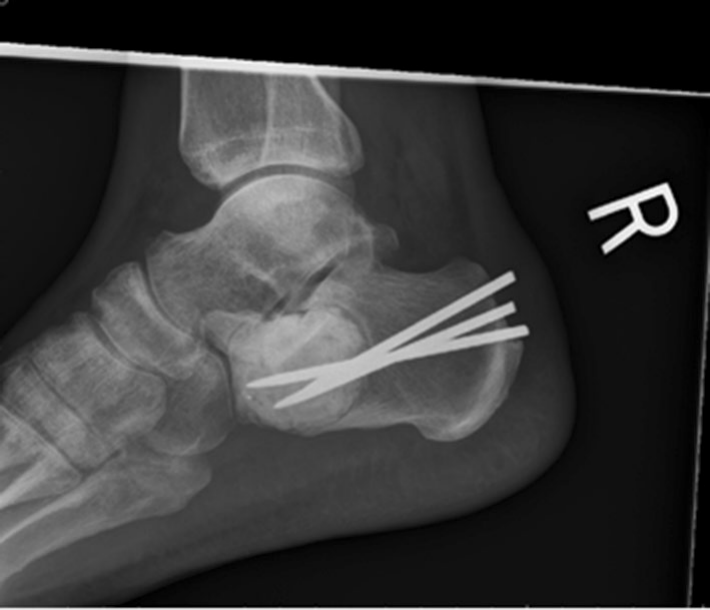
Two-year post-operative follow-up radiograph of the calcaneus showing curettage and cementing with Steinman pin reinforcement. There has been so sign of recurrence of disease or failure of the construct despite full activity

The patient was taken to the operating room for excision and curettage with argon beam followed by cement filling of the lesion and percutaneous pinning. A biopsy taken during surgery showed the tumor to be histologically the same as the previous biopsy, but now involving the surrounding bone. A thorough oncology and pulmonary workup for the mediastinal adenopathy ruled out metastatic disease, as a biopsy showed a benign anthracotic lymph node with marked histiocytosis. The patient returned to weight bearing activity at 3 months and work after 6 months. CT scan and radiographs of the hindfoot at his 6 month and 3 year follow-up showed well fixed hardware without recurrence (Fig. 10). PET scan at 3 years illustrated stable hilar lymphadenopathy and absence of enhancement of the calcaneus. Other than tolerable pain with extended walking, the patient has made full and tumor free recovery at 3 years.

## Discussion and conclusions

The majority of EHE cases of the bone occur in the lower extremities, followed by the spine [13]. The most common locations in the lower extremity for EHE to occur, in descending order, are the femur, tibia, fibula, and small bones of the feet [4]. Review of the current literature found four cases where the calcaneus was involved; however, none were unicentric. A case presented by Bisbinas et al. [14] described multicentric EHE of the foot and ankle in a 41-year-old male who presented with left foot and ankle pain. Plain radiographs showed multiple osteolytic lesions in the left lower extremity and CT showed no visceral involvement. The patient underwent excision and curettage of all lesions except for one that involved a rib. Biopsy showed no features of malignancy and the patient was alive with no further progression at 5.5 years.

The second case, reported by Liu et al. [5], described another multicentric presentation of EHE in the same lower extremity. The patient was a 59-year-old female who presented with swelling, pain, and functional deficits of her left lower leg. Physical examination demonstrated a 2.0 cm mass on the posterolateral aspect of the proximal lower leg and radiographs illustrated osteolytic lesions. CT showed involvement of the proximal fibula, distal tibia, cuneiform bone, and calcaneus. The patient was treated with fibular head resection and curettage of all other lower extremity lesions, followed by chemical cautery with a phenol solution. There was no local recurrence at 28 months after surgery and no evidence of internal metastasis.

A case report of a 17-year-old male illustrated another multicentric involvement of the lower extremity [18]. This young man presented with persistent pain of his right ankle and radiographs showed multiple bony lesions of the right lower extremity. CT identified lesions in the distal tibia, talus, calcaneus, and first cuneiform with some calcifications. The diagnosis of EHE was established with open biopsy and the patient received radiation therapy of 4600 cGY. No follow-up was reported.

The final case showed involvement of the calcaneus in a 28-year-old woman who presented with a 3-month history of left heel pain made worse by walking [16]. Radiographic imaging revealed a “lytic polylobulated lesion in the left calcaneus” which was not present on plain radiographs 2 months earlier. At a 3-month follow-up, the lytic defect showed slight expansion and an increase in the sclerotic reaction of surrounding bone. CT showed numerous small nodules in both lungs; however, there were no correlative complaints. The patient received adequate resection of the calcaneal lesion only. At 1 year follow-up, CT and MRI showed five small asymptomatic hepatic peripheral nodular lesions with no change in the pulmonary lesions.

As illustrated in our case and the above case reports, the most frequent clinical presentation of EHE of the bone is local pain [6–9]. Swelling is less commonly noted in the region of the lesion [9]. Radiographic imaging typically shows lytic lesions with a mix of features including surrounding sclerosis [4, 9], cortical destruction and expansion [4, 8, 9], and soft tissue expansion [4, 6, 8, 9]; however, there are no pathognomonic features that can be used to make the diagnosis. It is interesting to note the lack of radiographic evidence upon initial presentation in our case and in the aforementioned 28-year-old female, despite the presence of symptoms persisting greater than 2 months illustrating the *variable* nature of the malignancy. Clinically, these patients presented with an indolent clinical course; however, the rapid progression over a few months from negative radiographic identification to prominent lytic lesions suggests a rapid cellular development of the malignancy.

Microscopically, these lesions arise from vessels by expanding the vessel in a centrifugal pattern from the lumen extending outward into soft tissue [3, 7]. It has been shown that the majority of cases of EHE arise from veins [3, 7]. The tumor consists of round or cuboid epithelioid endothelial cells with abundant eosinophilic cytoplasm [9] embedded in a loose, lightly basophilic myxoid background [8]. In most cases, there is virtually no mitotic activity. In roughly one quarter of the cases, the “tumor contains areas with significant atypical, mitotic activity (more than 1 mitosis per 10 High Power Field), focal spindling, or necrosis” [3]. The most characteristic feature is the presence of intracytoplasmic vacuoles reminiscent of primitive lumen formation that may contain erythrocytes [4, 8]. This characteristic helps to distinguish it from epithelioid hemangioma and angiosarcoma, as each display well-formed vascular channels [8]. Immunohistochemical staining for CD31, CD34, vimentin, and FLI-1 help identify the vascular origin as the tumor can often be mistaken for metastatic carcinoma [7, 9].

Often the histological features can deviate from what is typical and show a solid growth pattern, epithelioid change, or spindle cell morphology. These changes can complicate the diagnosis. In 2012, Errani et al. [6] reported molecular analysis of 17 cases of EHE at different anatomical locations. They found an “identical genetic translocation [t(1, 3)(1p36.23;3q25.1)] involving the CAMTA1 and WWTR1 genes on chromosomes 1 and 3, respectively”. In 2014, Flucke et al. [12] identified the WWTR-1CAMTA1 fusion gene in 33 of 35 cases of varying anatomical EHE. The remaining two cases were found to contain a second fusion protein, YAP1-TFE3. All cases were identified using either fluorescent in situ hybridization (FISH) and/or reverse transcriptase-polymerase chain reaction (RT-PCR) [12].

There have been no predisposing factors identified [3]. Despite the variable nature of EHE, overall survival is high. In 1996, Kleer et al. [9] reported an overall survival of 89% for unicentric disease and 50% for multifocal disease. In a study of EHE in 62 patients in 2014, Angelini et al. found that 97% with unicentric disease lived to 10 years compared to 74% with multifocal disease [13]. From their study, Angelini et al. encourage using the type of presentation (unicentric vs. multifocal) in conjunction with degree of differentiation of histological features to determine prognosis [13]. Other studies state that atypical histological features (mitotic activity greater than 3/50 per high power field) and tumor size greater than 3 cm, regardless of anatomical location, have a higher rate of mortality [12, 19]. However, 10–15% of cases with histologically “benign appearing EHE metastasize and cause the death of the patient” [3]. Metastasis has been shown to occur in up to 31% of cases and is more common in those with marked cellular atypia, increased mitotic activity, spindling and necrosis [3, 4].

Complete and wide local excision is the proposed method of treatment [3–6, 9, 13]. Chemotherapy has been used for multifocal and metastatic cases [4, 5, 11–13] and radiation therapy alone or as an adjuvant has been successful in multicentric cases [4–6, 9, 13]. Although radiation can be successful, it should be reserved for cases not amenable to surgery as it has been shown to induce osteosarcoma [13].

## Abbreviations

CT: computed tomography
EHE: epithelioid hemangioendothelioma
EMA: epithelial membrane antigen
FISH: fluorescent in situ hybridization
MCK: mixed cytokeratin stain
MRI: magnetic resonance imaging
PET/CT: positron emission tomography/computed tomography
RT-PCR: reverse transcriptase-polymerase chain reaction
